# The Amount of Fluid Intake among Pregnant Women in China Increases with Pregnancy Progression: A Prospective Cohort Study

**DOI:** 10.3390/nu15224720

**Published:** 2023-11-08

**Authors:** Yongye Song, Fan Zhang, Xing Wang, Guotian Lin, Limin He, Zhixiong Lin, Na Zhang, Guansheng Ma

**Affiliations:** 1 Department of Nutrition and Food Hygiene, School of Public Health, Peking University, 38 Xue Yuan Road, Haidian District, Beijing 100191, China; songyongye@bjmu.edu.cn (Y.S.); mags@bjmu.edu.cn (G.M.); 2 International School of Public Health and One Health, Hainan Medical University, 3 Xue Yuan Road, Longhua District, Haikou 571199, China; zhangfan@hainmc.edu.cn (F.Z.);; 3 School of Health Medicine, University of Sanya, 191 Xue Yuan Road, Jiyang District, Sanya 572022, China; 4 Department of Pediatric Internal Medicine, Haikou Hospital of the Maternal and Child Health, 6 Wen Tan Road, Guo Xing Avenue, Qiongshan District, Haikou 570203, China; 5 Laboratory of Toxicological Research and Risk Assessment for Food Safety, Peking University, 38 Xue Yuan Road, Haidian District, Beijing 100191, China

**Keywords:** fluid intake, hydration status, pregnant women, prospective cohort study

## Abstract

Fluid intake and hydration status during pregnancy may have influences on maternal and infant health. However, few studies have recorded and analyzed total fluid intake (TFI) levels during the whole pregnancy. This study mainly aimed to investigate the TFI levels of pregnant women in three trimesters, and further to assess their hydration status. The relationships of TFI and hydration status were also analyzed. A convenience sampling method was used to recruit pregnant women from the Haikou Maternity and Child Health Hospital in China in this prospective cohort study. A 7-day 24 h fluid intake questionnaire was used for recording the fluid intake of the participants in their three trimesters. Fasting blood samples and first morning urine samples were also collected and tested. Hydration status was evaluated using urine osmolality. Finally, 142 pregnant women completed the study. The median TFIs in the first, second, and third trimesters were 1336, 1477, and 1584 mL, respectively. The TFI levels increased with pregnancy progression (χ^2^ = 134.155, *p* < 0.05). Out of 142 participants, 100.0%, 97.2%, and 85.2% of participants did not reach the recommendation amount for an adequate TFI among Chinese pregnant women in the three trimesters, respectively (χ^2^ = 29.840, *p* < 0.05). Plain water was the main source of fluid intake, accounting for 92.0%, 94.2%, and 93.4% of TFI, respectively. The median values of dairy product intake were 61, 57, and 59 mL in the three trimesters. The frequency of participants without an optimal hydration status in the three trimesters was 71.8%, 76.1%, and 83.1%, respectively (χ^2^ = 29.909, *p* < 0.05). The participants of each trimester were divided into four groups according to quartiles of TFI, including participants with a lower fluid intake (LFI_1_ and LFI_2_) and higher fluid intake (HFI_1_ and HFI_2_). As the TFI values increased from the LFI_1_ group to the HFI_2_ group, the urine osmolality decreased (all *p* < 0.05). Moderate-intensity negative correlations were found between urine osmolality, hydration status, and TFI (all *p* < 0.05). It is suggested that fluid intake strategies should be promoted and health education should be conducted to improve the hydration status of pregnant women.

## 1. Introduction

Water is a major component of the human body, accounting for 60~70% of its weight [1,2]. It acts as a medium to support the body’s biochemical reactions, and participates in multiple processes of metabolism. The maintenance of fluid and electrolyte balance is essential for health living [3]. Water is involved in maintaining a normal osmolality, electrolyte balance, and body temperature [2]. Sources of water intake include fluid intake from drinking water and beverages, water intake from food, and water produced by oxidative processes in the human body [4].

Pregnancy is a physiological condition in which increases in body weight and composition occur during a short period [5]. A woman’s body undergoes a series of changes throughout gestation to meet the needs of fetal development and safe delivery, including the blood system, urinary system, and digestive system [6]. This leads to an accretion in total body water content, which presents exceptional challenges to water homeostasis during pregnancy. Gestational body weight gain includes fat deposition, total body water buildup, and the growth of products of conception [5]. Under healthy conditions, a woman gains 12.5 kg in weight throughout pregnancy, with liquid gain accounting for 6~8 kg [7]. Blood volume increases from 6 weeks’ gestation and reaches a peak from 4700 to 5200 mL at 32 weeks’ gestation. In particular, at term, the total blood volume increases by 1.4~1.8 kg [8,9,10]. The water excretion of the body also increases during pregnancy. Furthermore, the hormone levels of pregnant women will increase, including progesterone, estrogen, and prostaglandins. As early as 5 weeks gestation, maternal cardiac output increases [11]. The glomerular filtration rate increases by 25% at 2 weeks’ gestational age and 50% by the early second trimester, which is 50~80% higher than that in non-pregnancy [12,13,14]. In addition, the tidal volume and minute ventilation of pregnant women can increase by about 50%, which leads to an improvement in the water level excreted through respiration [15]. Sweating through the skin is one of the paths for water excretion, as approximately 500 mL water would evaporate through the skin every day. During pregnancy, the adrenal and thyroid functions are hyperactive, leading to an increased skin blood circulation and accelerated metabolism. Thus, water output caused by sweating from the skin also increases. It is worth noting that morning sickness in the first trimester of pregnancy is also one of the important reasons for breaking the water balance during pregnancy. A previous study found that more than 50% of pregnant women have experienced nausea and vomiting in the first trimester of pregnancy, among whom, 70~80% indicated that they were influenced by these symptoms [16]. Therefore, the demand for water during pregnancy increases compared to during non-pregnancy. This leads to a requirement for a higher fluid intake level to maintain the balance of body water content.

A normal hydration status is the condition of healthy individuals who maintain their balance of water input and output [17]. An insufficient fluid intake would lead to dehydration. Extensive evidence has shown that dehydration may reduce the ability of cognitive performance [18,19] and physical activity [20,21], and have negative influences on kidney excretion [22,23] and the nervous system [24,25]. On the one hand, fluid intake during pregnancy can have an impact on maternal health. Low fluid intake has been linked to constipation in pregnancy, particularly in the third trimester [26]. A previous study revealed that dehydration in pregnant women could also lead to oligohydramnios [27]. On the other hand, evidence of an association between fluid intake and risk of adverse pregnancy outcomes has been found. In a case–control study conducted in America on 2591 women, the results showed that, compared to those with water intake levels of 1~3 glasses per day, pregnant women whose water intake level reduced had an increased risk of premature birth and abortion. However, the risk decreased with water intake level improving [28]. In addition, previous studies have revealed that a normal amniotic fluid volume is vital for fetal growth and development, which could be a predictor of fetal health [29]. Another study conducted in Canada determined the body composition of 196 women 4~12 h after delivery [30]. The result showed that their average level of total body water content was 34.8 L, which explained a major proportion of the variability in birth weight in comparison to maternal weight gain during pregnancy. Thus, total body water content has already been considered as an important predictor of birth weight. Furthermore, it cannot be ignored that the water intake levels of women during pregnancy have a long-term effect on their infant’s early development. A prospective cohort study conducted in China on 985 women and their infants showed that a high sugar-sweetened beverages (SSBs) intake level during pregnancy was associated with an increased risk of delay in the social–emotional development of children at 6 and 12 months old [31].

Regarding the influences caused by an insufficient fluid intake, some countries have developed recommendations for the adequate water intake of pregnant women, while these levels vary among different countries. The World Health Organization (WHO) noted in a report that the total water intake level that meets the requirement of pregnant women is 4800 mL per day [32]. The recommended adequate total water intake level for pregnant women by the American Medical Research Institute is 2700 mL/day, which is 300 mL/day higher than that of non-pregnant women [33]. As energy intake during pregnancy increases by 300 kcal per day, the European Food Safety Authority formulated the adequate total water intake level through calculations in 2010. The adequate total water intake level for European pregnant women is set as 2300 mL/day, 300 mL/day higher than that for adult women who are not pregnant [34]. The National Institute of Nutrition of Indian Council of Medical Research also suggested that the daily fluid intake of pregnant women in India should reach the level of 2100~3200 mL/day, which was calculated according to the weight and energy requirements of pregnant women [35]. The adequate water intake level was put forward in the Dietary Reference Intakes for Chinese Residents (2013) [36]. However, no fluid intake research was conducted on pregnant women in China at that time. The reference for increase in level of TFI during pregnancy was determined based on referencing the data from America. Considering the fluid intake patterns of Chinese people, based on the adequate water intake of healthy adult women who are not pregnant in China, the total water intake level and adequate TFI were set as 3000 mL/day and 1700 mL/day. However, many factors influence water requirements, including race, climate, and dietary culture. As a result, the reference based on the America data may not be suitable for pregnant women in China. Thus, it attaches great importance to performing related research to explore the fluid intake and hydration status of pregnant women in China.

In our present study, the primary aim was to track the TFI levels among women in different trimesters of pregnancy. The second aim was to determine urine and blood biomarkers, so as to evaluate hydration status of participants. Finally, the third aim was to explore the association between the TFI and hydration status of participants. The results of this study could provide valuable references for the TFI levels of pregnant women in China. Thus, hydration status can be further evaluated according to TFI levels.

## 2. Methods

### 2.1. Study Design

This was a prospective cohort study which was conducted from August 2019 to March 2021 in Hainan, China. The participants were pregnant women attending outpatient clinics at the Haikou Hospital of The Maternal and Child Health. Three follow-up visits were performed in three trimesters during pregnancy periods, respectively. A convenience sampling method was used for recruitment. Participants who met the inclusion criteria were enrolled in this present study.

### 2.2. Sample Size Calculation

In this prospective cohort study, the sample size was determined using the following formula: N = [1 + (K + 1)r]s^2^(Z_1−a/2_ + Z_1−b_)^2^/Kd^2^. The variable used was the fluid intake volume of pregnant women. In a previous study conducted in Indonesia, the standard deviation of daily fluid intake was 750 mL [6]. Z is the value associated with the desired confidence level, and the confidence level was set at 95% (Zα = 1.96) in our present study. In the above formula, s = 750, d = 200 [37], K = 3, α = 0.05, b = 0.8, Z_1−b_ = 1.28, and t = 1.96. Considering a dropout rate of 10%, at least 142 participants were needed.

### 2.3. Participants

**Figure 1:**
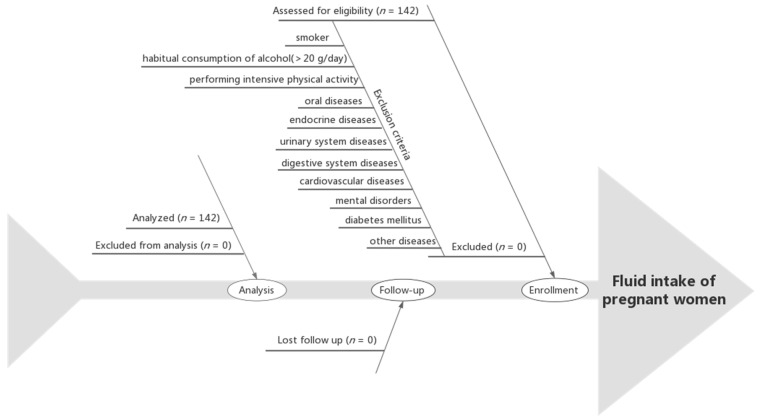
Participants flow chart.

The inclusion criteria were as follows: first prenatal examination prior to the 13th week of gestation; maternal age of 21~35 years at delivery; first pregnancy; singleton pregnancy; being in a healthy condition before enrollment; and performing regular prenatal examinations at the Haikou Hospital of The Maternal and Child Health. The exclusion criteria were as follows: an admission of tobacco or illicit drug use before or during pregnancy; habitual consumption of alcohol (>20 g/day) [38]; performing intensive physical activity; communication barriers; oral diseases, endocrine diseases, urinary system diseases, digestive system diseases, cardiovascular diseases, or mental disorders; diabetes mellitus; or other diseases prior to the pregnancy. The flow chart of the participants is shown in Figure 1. 

### 2.4. Ethical Standards

The study protocol was reviewed and approved by the Ethical Review Committee of the Hainan Medical University. The ethical approval project identification code is 2018-4. The study protocol was registered on the Chinese Clinical Trial Registry website under trial registration number Chi CTR 800019284. The study was conducted according to the principles of the Declaration of Helsinki. Prior to the beginning of the study, all the participants read and signed their informed consent forms in duplicate voluntarily.

### 2.5. Study Procedure

After the participants who met the inclusion criteria were recruited, their basic information was collected using a questionnaire prior to the 13th week of gestation. This was a prospective study and three follow-up visits were conducted during pregnancy. To eliminate the effect of morning sickness, the first visit was performed during 15~17 weeks of gestation for each participant. The second one and third one were carried during the second (20~22 weeks of gestation) and third (30~32 weeks of gestation) trimesters of pregnancy, respectively. Correspondingly, 7-day 24 h fluid intake surveys, blood sample collections, and urine sample collections were conducted in the three trimesters. A 7-day 24 h fluid intake questionnaire was used to record the fluid intake behaviors of the participants from day 1 to day 7 in each trimester. The participants were required to record their behaviors in real time and in free-living conditions [39]. The participants’ TFIs were evaluated and recorded using a standardized customized cup. The questionnaires were collected and checked on day 8 of each follow-up visit by researchers. The urine collectors were sent to the participants before their prenatal examination. The first morning urine was collected by themselves and carried to the hospital. The urine samples were packaged according to the test items and tested within 2 h. The fasting blood samples were collected during their prenatal examinations and stored in an environment of −20 °C. Anthropometric measurements were performed on day 8 in each follow-up visit, including height, weight, skeletal muscle, body fat rate, body water content, and blood pressure. Environmental parameters during the study period were recorded from day 1 to day 7 at each follow-up visit. The indicators collected at different study time points in each trimester are presented in Table 1.

### 2.6. Anthropometric Measurement

Height and weight: The height and weight of the participants were measured in the first, second, and third follow-up visits during pregnancy, respectively. A height–weight meter (DHM-300; Huaju, Yiwu, Zhejiang, China) was used to measure the height and weight of the participants. Each measurement was performed twice by uniformly trained investigators following the standardized methods. The average values were calculated and recorded. In addition, participants’ BMI (body mass index) values were calculated (BMI: weight (kg)/height squared (m)).

Blood pressure: The blood pressure of the participants was measured in the first, second, and third trimesters during pregnancy, respectively. Blood pressure was measured by uniformly trained investigators using an upper-arm electronic sphygmomanometer (U10L; Omron, Dalian, China). The measurement in this study was conducted according to the standard process [40]. The participants were required to avoid any intense exercise or the consumption of any beverages containing caffeine for one hour before the measurement. They were required to sit in chairs with their body relaxed and heavy clothing removed. The systolic and diastolic blood pressures were determined according to the Korotkoff sound. Two consecutive measurements were obtained at 2 min intervals and the average values of both the systolic and diastolic blood pressure were taken and reported in this study.

Skeletal muscle: The body mass of the participants was measured three times in the follow-up visits, respectively. The body composition of the participants was measured by investigators using a body composition analyzer (Inbody 720; Inbody; Seoul, Republic of Korea). They were in fasting state before the measurements and after having defecated and urinated, wearing light clothes with their metal jewelry removed. Meanwhile, their feet had to be in a clean and dry status. The participants’ information was input into the instrument, including age, gender, and height. After the screen was reset to zero, the participants stood on the instrument. Their palms and plantar were contacted with the electrode surface, keeping them upright and maintaining an angle of approximately 30° between their body and upper limbs. The participants were required to stay still during the process. When the measurements were completed, two measurement results were recorded and the average values were calculated.

Body fat rate and body water content: These two items were measured using the body composition analyzer (Inbody 720; Inbody; Seoul, Republic of Korea) in the above process.

### 2.7. Assessment of TFI Level

The daily total water intake is the sum of the TFI (the fluid intake from water and beverages, accounting for 50% approximately) and daily water intake from food (accounting for 40% approximately) [41]. The TFI is the total volume of fluid intake from water and beverages, excluding water from food. In this study, the water intake levels from food were not recorded or measured.

On the basis of the questionnaire used in previous studies conducted in China, a standardized and verified “7-day 24 h fluid intake questionnaire” was used for the fluid intake record [42,43,44,45]. The participants’ fluid intake levels and types in each trimester for 7 consecutive days were recorded by themselves in detail. According to the standard in China, the types of fluid comprised plain water, dairy products, and sugar-sweetened beverages (SSBs) [46]. Plain water included tap, packaged, and mineral water in this study. Dairy products referred to pure milk, yogurt, and any other dairy products with no sugar added during production. SSBs referred to beverages with sugar added during processing, including carbonated, fruit and vegetable juice, protein, coffee, plant-based, flavored, and special-purpose beverages. Each participant was provided with a customized cup, which was used to assist in estimating the amount of fluid intake each time. The capacity of the cup was 400 mL, with a closest scale of 10 mL. The participants were required to conduct three real-time fluid intake records for 7 consecutive days in the study period of the first, second, and third trimesters of pregnancy, respectively. After completing the record each time, the questionnaires were collected and checked by investigators.

### 2.8. Tests Conducted for Urine Biomarkers

The urine sample collections were conducted three times during the study, in the first, second, and third trimesters of pregnancy, respectively. The first morning urine samples were collected in sterile disposable urine sample cups and stored in a 4 °C thermostat.

Urine osmolality: An osmotic pressure molar concentration meter (SMC 30C; Tianhe, Tianjin, China) was used to test the urine osmolality with the freezing point method. The test was performed in light of the Standard Operating Procedure for Determination of Osmolality Molar Concentration for Chinese Drug Testing Standard Practice 2016 Edition.

Urine-specific gravity (USG): A fully automatic urinary analyzer (HC-900; Dirui; Changchun, China) based on the uric dry-chemistry method was used for determination. Test strips filled with urine samples were pushed into the testing area for determination. The results displayed on the analyzer were recorded.

Urine pH: A fully automated urine analyzer (HC-900; Dirui; Changchun, China) was used to determine the pH of the urine samples based on the acid-base titrimetric method. Indicators presented in different colors contacted with urine samples of different pHs. The results displayed on the analyzer were recorded.

### 2.9. Judgment and Definition of Hydration Status

Referring to previous studies [44,47], the hydration status of the participants was assessed and classified according to the urine osmolality. The hydration statuses of the participants were divided into three groups according to the levels of urine osmolality: groups with a dehydrated status, normal hydrated status, and optimal hydrated status. A dehydrated status was defined as a urine osmolality of >800 mOsm/kg [47,48,49]. A normal hydrated status was defined as a urine osmolality of ≤800 mOsm/kg but >500 mOsm/kg [17]. An optimal hydrated status was defined as a urine osmolality of ≤500 mOsm/kg [50].

### 2.10. Determination of Blood Biomarkers

Blood sample collection was performed three times during the first, second, and third trimesters, respectively. A volume of 2 mL of fasting blood from antecubital venous was collected from the participants in the morning each time.

Blood osmolality: An osmolality weight molar concentration tester (BS800; Mindray; Shenzhen, China) was used to measure the blood osmolality. The tests were in accordance with the Standard Operating Procedure for Determination of Osmolality Molar Concentration for Chinese Drug Testing Standard Practice 2016 Edition.

Blood glucose: An automatic biochemical analyzer (AU5800, Beckman, Brea, CA, USA) was used to test the blood glucose levels via spectrophotometry.

Other blood biomarkers: Blood was collected in vacuum tubes and serum was separated via centrifugation. An automatic biochemical analyzer (BC5180; Mindray; Shenzhen, China) was used for the detection of the presence of hemoglobin, red blood cells, white blood cells, lymphocytes, and platelets.

### 2.11. Temperature and Humidity in the Environment

The data of the daily minimum and maximum temperatures in Haikou during the study periods were recorded from the China Meteorological Administration. The temperature of the day was defined as the median temperature, and then the average temperatures during the study periods were calculated. In addition, the humidity for 7 consecutive days during each follow-up visit periods was also recorded.

### 2.12. Quality Control

Prior to the study, a unified procedure was developed for three follow-up visits. The questionnaire was formulated before the study based on a literature review, focus group discussion, and expert consultation.

A research manual was developed for the study, including a research protocol, questionnaire, methods, and procedures. Training was conducted among the investigators before the study, including the investigation methods and data verification methods. The participants were trained on the questionnaire records. Equipment and instruments of the same model were used in the three follow-up visits. Effective and convenient communication methods were used to improve follow-up rates. Furthermore, data quality was guaranteed by home visits and phone calls performed between the participants and investigators. During the whole research process, the investigators conducted strict supervision over all procedures. Double checking was performed on the completed questionnaires every day. Before the data input, each item in the questionnaire was encoded and checked, and then erroneous items were cleared.

### 2.13. Statistical Analysis

The database was established using the EpiData 3.1 software. A double-entry method was applied for checking and cleaning up wrong items promptly. The data analysis was assessed using the SPSS Statistics 26.0 (IBM Crop., Armok, NC, USA). Normality tests were conducted on the data. The results were reported as mean ± standard deviation (SD) for normally distributed data. The results were reported as median and quartile ranges (M and Q) for abnormally distributed data. In addition, the data were presented as the mean number of participants according to the diagnostic criteria. A one-way ANOVA was used for comparing the differences in the normally distributed data (reported as mean ± SD) among the four groups, such as the age, height, weight, BMI, skeletal muscle, body fat rate, body water content, and blood pressure. The abnormal distribution data (shown as M and Q) were compared using a Kruskal–Wallis H-test among the four groups. The proportions of participants among the four groups were compared using the Chi-square test. The Student–Newman–Keuls (SNK) method (*p* < 0.05) was used for comparing the differences between each two groups. A Friedman test was used for comparing the differences of fluid intake, urine indexes, blood indexes, and hydration status between the first, second, and third trimesters of pregnancy. Furthermore, the intensity of the correlations among urine biomarkers, blood biomarkers, hydration status, and fluid intake was analyzed using Spearman’s correlation coefficients. The a-level was set at 0.05 for statistical significance (*p* < 0.05).

## 3. Results

### 3.1. Participants’ Characteristics and the Temperature

In this study, a total of 142 participants who met the inclusion were recruited. Finally, 142 participants completed the study, which was a 100% completion rate. The characteristics of these 142 participants are presented in Table 2.

The participants in each trimester were divided into four groups, LFI_1_ (low fluid intake 1), LFI_2_ (low fluid intake 2), HFI_1_ (high fluid intake 1), and HFI_2_ (high fluid intake 2), according to the quartiles of TFI in the first trimester (Q_1_: 1096~1254 mL, Q_2_: 1255~1336 mL, Q_3_: 1337~1437 mL, and Q_4_: 1438~1639 mL), second trimester (Q_1_: 1220~1401 mL, Q_2_: 1402~1477 mL, Q_3_: 1478~1560 mL, and Q_4_: 1561~1836 mL), and third trimester (Q_1_: 1200~1510 mL, Q_2_: 1511~11,584 mL, Q_3_: 1585~1649 mL, and Q_4_: 1650~1950 mL), respectively. The factors of age, height, weight, BMI, skeletal muscle, diastolic pressure, and systolic pressure did not differ significantly between the four groups in any trimester (all *p* > 0.05). However, the body fat rate and body water content differed significantly between the four groups in the second trimester (*F* = 3.439, *p* < 0.05, *F* = 4.209, *p* < 0.05).

The average temperature calculated was 27.6 ± 3.3 °C and the average humidity was 78.3 ± 7.9% RH during this period in Hainan.

### 3.2. Records of TFI of Participants at Different Trimesters of Pregnancy

The daily TFI levels in the three trimesters of pregnancy were measured in the present study. The results are shown in Table 3. Among the 142 participants, the TFI levels differed significantly in the three trimesters, as the median values were 1336, 1477, and 1584 mL, respectively (χ^2^ = 134.155, *p* < 0.05). The participants in the third trimester had the highest TFI levels. The percentages of participants who did not meet the recommendation of an adequate water intake level in three trimesters (1700 mL for pregnant women in China) were 100.0%, 97.2%, and 85.2%, respectively. This also differed significantly in the three trimesters (χ^2^ = 29.840, *p* < 0.05). Plain water accounted for 92.0%, 94.2%, and 93.4% of daily TFI, respectively, which was the main source of fluid intake of the participants. There were significant differences in participants’ plain water intake levels between three trimesters (χ^2^ = 149.534, *p* < 0.05). The median values of dairy product intake were 61, 57, and 59 mL in the three trimesters, which exhibited no significant differences (*p* > 0.05). These participants also had low intake levels of dairy products and SSBs.

### 3.3. Tests of Urine Biomarkers of Participants at Different Trimesters of Pregnancy

The data in Table 4 revealed that, with an increase in the TFIs, the osmolality of urine decreased from the LFI_1_ to HFI_2_ groups and was significantly different between the four groups in three trimesters (χ^2^ = 46.197, *p* < 0.05; χ^2^ = 26.728, *p* < 0.05; and χ^2^ = 15.298, *p* < 0.05). Out of 142 participants, 28.2%, 23.9%, and 16.9% of the participants had an optimal hydration status in the three trimesters, respectively, which was evaluated with the urine osmolality. The percentage of participants who had a dehydrated status decreased from 18.3% in the first trimester to 1.4% in the third trimester of pregnancy (χ^2^ = 29.909, *p* < 0.05). The median values of USG (urine-specific gravity) were 1.015, 1.020, and 1.015 in the three trimesters, respectively, while there were no significant differences (*p* < 0.05).

There were significant differences in the hydration status between the participants of the four groups in each trimester. During the first trimester, the USG, urine pH, and urine creatinine values differed significantly between the four groups (χ^2^ = 9.591, *p* < 0.05; χ^2^ = 12.138, *p* < 0.05; and χ^2^ = 11.295, *p* < 0.05). During the second trimester, there existed significant differences between the USG values of the four groups (χ^2^ = 8.847, *p* < 0.05). During the third trimester, the urine creatinine and urine acid values differed significantly among the four groups (χ^2^ = 12.995, *p* < 0.05; χ^2^ = 8.503, *p* < 0.05). There were no significant differences between urine osmolality, hydration status, USG, urine pH, urea, urine creatinine, or urine acid in different trimesters (all *p* > 0.05) (Table 4).

### 3.4. Tests of Blood Biomarkers of Participants at Different Trimesters of Pregnancy

There existed significant differences between the concentration levels of TG and LDL of the participants in the four groups during the first trimester (χ^2^ = 9.264, *p* < 0.05; χ^2^ = 10.005, *p* < 0.05). No statistically significant differences were observed in the concentration levels of blood glucose, blood lipids, white blood cell count, red blood cell count, hemoglobin, hematocrit, mean red blood cell volume, mean red blood cell hemoglobin content, platelet content, serum protein, serum bilirubin, and lymphocyte count among the participants of the four groups in the second and third trimesters (all *p* > 0.05). The concentration levels of blood glucose, LDL, and lymphocyte count of the participants in different trimesters of pregnancy differed significantly (*F* = 122.187, *p* < 0.05; *F* = 8.646, *p* < 0.05; and *F* = 6.233, *p* < 0.05) (Table 5).

### 3.5. Correlations between Fluid Intake Behaviors, Hydration Status, Urination Biomarkers, and Blood Indexes

Regarding the first trimester, moderate-intensity negative correlations were found between urine osmolality, hydration status, and TFI (*r* = −0.596, *p* < 0.05; *r* = −0.570, *p* < 0.05). Weak-intensity negative correlations were found between USG, LDL, and TFI (*r* = −0.180, *p* < 0.05; *r* = −0.261, *p* < 0.05). Weak-intensity negative correlations were found between urine osmolality, USG, LDL, and plain water (*r* = −0.312, *p* < 0.05; *r* = −0.167, *p* < 0.05; and *r* = −0.312, *p* < 0.05). Strong-intensity negative correlations were found between hydration status and plain water (*r* = −0.716, *p* < 0.05) (Table 6).

Regarding the second trimester, moderate-intensity negative correlations were found between urine osmolality, hydration status, and TFI (*r* = −0.439, *p* < 0.05; *r* = −0.410, *p* < 0.05). Meanwhile, moderate-intensity negative correlations were found between urine osmolality, hydration status, and plain water (*r* = −0.418, *p* < 0.05; *r* = −0.349, *p* < 0.05).

Regarding the third trimester, strong-intensity negative correlations were found between hydration status and TFI (*r* = −0.444, *p* < 0.05). Weak-intensity negative correlations were found between urine osmolality, urine creatinine, urine acid, and TFI (*r* = −0.295, *p* < 0.05; *r* = −0.252, *p* < 0.05; and *r* = −0.230, *p* < 0.05). Meanwhile, moderate-intensity negative correlations were found between urine osmolality, hydration status, urine creatinine, urine acid, and plain water (*r* = −0.213, *p* < 0.05; *r* = −0.368, *p* < 0.05; *r* = −0.253, *p* < 0.05; and *r* = −0.269, *p* < 0.05).

These biomarkers whose values did not differ significantly between the four groups were not analyzed for correlations.

## 4. Discussion

In the present study, we investigated fluid intake levels and types in different trimesters during pregnancy with the method of tracing a prospective cohort of pregnant women. Furthermore, related urine biomarkers, hydration status, and blood biomarkers were measured to explore the effect of fluid intake on health. The results obtained in this study demonstrated that pregnant women had an insufficient fluid intake in different trimesters. The data collected in this study revealed that the median TFI values of the participants in the first and second trimesters were 1336 and 1477 mL, respectively, which were both lower than the 1700 mL per day recommended by the Chinese Dietary Guidelines (2022) for pregnant women [51]. The values were even lower than the 1500 mL per day recommended by the Chinese Dietary Guidelines (2022) for non-pregnant adult women. The median TFI of the participants in the third trimester was 1584 mL, which was also lower than the 1700 mL recommended per day for pregnant women. On the whole, totals of 100.0%, 97.2%, and 85.2% of participants did not meet the recommended level in their first, second, and third trimesters, respectively.

The present and previous studies have shown that an insufficient fluid intake among pregnant women is widespread. A study conducted in Indonesia showed that about 42% pregnant women and 54% breastfeeding women, respectively, had an insufficient water intake and this level was under the recommendation [6]. Another study conducted in China on 583 pregnant women in 2020 showed that the median TFI was 1321 mL, and the plain water intake level was the highest (1000 mL), followed by dairy products (179 mL) and other beverages (29 mL). The daily TFI levels of the participants in the first, second, and third trimesters of pregnancy were 1000 mL, 1457 mL, and 1446 mL, respectively [52]. These were close to but lower than the results obtained in our present study. This is possibly due to the different methods used in the two studies. Retrospective questionnaires were used in previous studies and fluid intake volumes were recorded based on participants’ self-estimations by referring to pictures of cups with a scale. In contrast, a 7-day 24 h real-time fluid intake record and cups with a scale were used to measure the fluid intake volume each time in our present study. These can avoid the inaccuracy of recall and estimation. A previous study compared the TFI value assessed with a 7-day fluid record versus the value from 24 h dietary recall in Indonesia among adolescents and adults. It manifested that the 24 h dietary recall underestimated TFI with a bias of up to 382 mL/day [53,54]. Thus, compared to other methods, the 7-day 24 h record has frequently been used in many nutrition surveys [55,56]. A study carried out on 653 pregnant women in China showed that the median TFI was 1165 mL. This values is higher than that found in our present study [57]. This difference might be related to the geographical location of the participants, as Hainan is a tropical island along the southern coast of China. Higher temperatures led to an increase in fluid intake. Compared to the results obtained from previous studies, the fluid intake level of the pregnant women in this study was higher than that of non-pregnant women in China. A study conducted in 2016 investigated the fluid intake levels of 2233 participants from 27 cities in China. The results showed that the daily TFI values of adults women were 1332 mL, which did not meet the adequate water intake level in China [58]. Another study conducted on 156 college students in Hebei, China, revealed that the daily TFI level of female college students was 958 mL [59]. The possible reason for differences between the fluid intake levels of pregnant women and non-pregnant women may be associated with the increased demand for water during pregnancy. On the one hand, pregnant women’s blood volume increases, which requires more water to regulate the body fluid balance. On the other hand, a series of physiological changes occur during pregnancy, including an increased glomerular filtration rate, accelerated skin blood circulation, increased tidal volume, and minute ventilation. As a result, these lead to an increase in water loss through urination, sweating, and breathing [13,15].

It is worth noting that the TFI increased significantly with pregnancy progression (χ^2^ = 134.155, *p* < 0.05). This was consistent with the result in a study conducted on 232 pregnant women in France in 2014, which showed that the TFI in the third trimester (1937 mL/day) was higher than the TFI in the second trimester (1827 mL/day) [60]. However, in contrast to our results, two studies conducted in China reported no differences between TFI levels during pregnancy [61,62]. The inconsistency may have been caused by different methods. A pregnancy cohort was prospectively followed up in our study, which can reduce confounding bias, while a cross-sectional record was used in the previous one.

Plain water intake was the main source of fluid intake, which accounted for 92.0%, 94.2%, and 93.4% of the TFI in the first, second, and third trimesters of pregnancy, respectively. This was consistent with the results in previous studies [61,62]. In our study, the medians of the dairy product intake of the participants in the first, second, and third trimesters were 61, 57, and 59 mL, respectively, which were much lower than the recommended intake level in China (300 mL/day) [36]. The data obtained in our study were lower than the results reported in the previous one in China, which showed 86, 200, and 229 mL dairy product intake levels in the three trimesters, respectively [57]. In addition, the participants consumed low levels of SSBs (0~68 mL) in this study, which may have been due to their high attention to health during pregnancy. Accumulating evidence has linked SSB intake with an increased risk of pre-eclampsia, preterm delivery, and other pregnancy complications [31,62].

Assessed with the urine osmolality, the frequency of participants with a dehydrated status was 18.3%, 6.3%, and 1.4% in the first, second, and third trimesters, respectively. According to previous studies in other countries, dehydration may be common during pregnancy, which is caused by an insufficient fluid intake. A study conducted in Jakarta on 35 women at the second trimester of pregnancy reported that 57.1% of participants had a dehydrated status [63]. Another study conducted on 27 pregnant women who were overweight or obese showed that 49.1% individuals had a dehydrated status [64]. Therefore, great importance is attached to providing health education to correct fluid intake behaviors during pregnancy, so as to reduce the rate of dehydration.

Early studies demonstrated that urine osmolality and USG appeared to be a favorable approach for assessing renal function and hydration status [65,66]. Early in 1988, investigators suggested that reliable information about hydration status could be provided from a measurement of the first morning urine, which was a quick and easy method for accessing hydration status [67]. Compared to the urine osmolality (666 vs. 597 mOsm/kg, 672 vs. 514 mOsm/kg, and 659 vs. 497 mOsm/kg) and USG (1.015 vs. 1.014, 1.020 vs. 1.013, and 1.015 vs. 1.013) in the first, second, and third trimesters of pregnant women from Korea, the results of dehydrated rates in our study were at higher levels [68]. This may have been influenced by the urine samples at different time points, as first morning urine samples were determined in our study, while 24 h samples were collected in the previous one. There were daily fluctuations in urine production influenced by the circadian pattern of arginine vasopressin release. The urine osmolality and the concentration level of urine biomarkers also fluctuated at different time points [69,70]. Moreover, the study conducted in Korea found decreases in urine osmolality and USG values during pregnancy [71]. Another study conducted in America found that an inverse-U shape relation was observed with urine osmolality. Compared to the value in the first trimester, it increased in the second trimester, while it fell in the third trimester [64]. However, no trend of any urine or blood biomarkers related to hydration status was found in our study, which was similar to the results of studies in Thailand and Canada [65,71]. Considering the long interval of follow-up visits in this study, subtle changes were difficult to capture, which may have resulted in some biases. Our result found that a higher plain water intake level was associated with a higher TG concentration and lower LDL concentration. Some previous finding was that water intake and hydration were beneficially associated with circulating lipid concentrations. These findings were supported by observations that a better hydration was associated with lower TG and LDL cholesterol and higher HDL cholesterol concentrations. But the result from our study was inconsistent with previous studies [72,73]. A possible reason was that the hydration status (assessed by 24 h urine creatinine concentrations) was used to analyze the association, while the factor analyzed in our study was fluid intake. Total water intake includes fluid intake and water intake from food, while the latter provides a high level of water intake. Thus, the effects of fluid intake and hydration status on TG concentration and lipids concentration need to be explored in detail comprehensively in the future.

Several strengths and limitations in our present study can be identified. In terms of strengths, this is the first study to prospectively evaluate the TFI levels in three trimesters during pregnancy in China. The method used in this prospective cohort study can avoid the bias caused by recalling time point data. Furthermore, fluid intake types were recorded, and the correlations between fluid intake types with hydration status were further analyzed. In addition, the method of 7-day 24 h fluid intake record in real-time was used in this study, which greatly improved the accuracy of the data. However, our study has some limitations. First of all, we only investigated the TFI levels of participants; however, the water intake from food was not recorded and analyzed. As a result, it cannot reflect the total water intake level of pregnant women comprehensively. Secondly, only morning urine was collected and tested, which may have led to some limitations to the evaluation of hydration status. As the 24 h urine osmolality was not determined, hydration status throughout the day cannot be reflected owing to urine circadian fluctuations [69]. In addition, the participants in this study were from Haikou, a city located at the southern end of China. Taking this into consideration, the TFI levels of the pregnant women may have been influenced by the local climate and temperature. Thus, the results of this study cannot be generalized for the whole Chinese pregnant population.

## 5. Conclusions

The TFI levels of pregnant women increased with pregnancy progression. However, the TFI levels were inadequate compared to the recommendation for the adequate intake of water for pregnant women (1700 mL per day). Plain water was the main source of fluid intake. Participants with a higher fluid intake level had a superior hydration status. The results can provide references for evaluating hydration status according to fluid intake level. It is suggested that water intake strategies should be developed and health education on fluid intake should be increased to improve the hydration status of pregnant women. In the future, studies that track the health status of pregnant women and their infants throughout the whole pregnancy are needed to explore the association of water intake and health.

## Figures and Tables

**Table 1:** The indicators and pregnancy outcomes collected at different time points in each trimester in this study.

	Prior to the 13th Week of Gestation	In the First Trimester		In the Second Trimester		In the Third Trimester	
		Day 1–Day 7	Day 8	Day 1–Day 7	Day 8	Day 1–Day 7	Day 8
Individual information	√						
Anthropometric measurement	√		√		√		√
7-day 24 h fluid intake questionnaire	√	√		√		√	
Blood biomarkers			√		√		√
Morning urine and related biomarkers			√		√		√
Environment		√		√		√	

Note: √ means that the measure has been taken on that period.

**Table 2:** Characteristics and daily TFI of participants in different trimesters of pregnancy.

	*N* (%) ^a^	Age (y) ^b^	Height (cm) ^b^	Weight (kg) ^b^	BMI (kg/m^2^) ^b^	Skeletal Muscle (kg) ^b^	Body Fat Rate (%) ^b^	Body Water Content (%) ^b^	Blood Pressure	
									Systolic (mmHg) ^b^	Diastolic (mmHg) ^b^
First trimester										
F-LFI_1_	38 (26.8)	28.9 ± 3.5	157.8 ± 4.9	52.6 ± 5.4	21.2 ± 2.3	38.9 ± 3.9	20.9 ± 2.6	46.1 ± 3.7	113.3 ± 8.8	73.4 ± 6.9
F-LFI_2_	36 (25.4)	27.5 ± 2.8	156.5 ± 4.7	50.1 ± 5.0	20.5 ± 1.7	37.8 ± 3.5	21.3 ± 2.9	46.6 ± 4.9	111.0 ± 8.8	72.0 ± 6.6
F-HFI_1_	33 (23.2)	28.2 ± 3.0	156.5 ± 4.5	50.1 ± 5.2	20.5 ± 1.9	38.3 ± 4.4	21.3 ± 3.3	46.6 ± 3.5	109.7 ± 9.1	70.0 ± 6.1
F-HFI_2_	35 (24.6)	29.3 ± 3.7	157.0 ± 4.3	50.0 ± 4.2	20.1 ± 1.5	37.8 ± 3.2	22.3 ± 3.0	47.9 ± 4.3	110.4 ± 10.7	71.4 ± 4.8
F-Total	142	28.5 ± 3.3	156.9 ± 4.6	50.7 ± 5.1	20.5 ± 1.9	38.2 ± 3.8	21.4 ± 3.0	46.8 ± 4.1	111.1 ± 9.4	71.7 ± 6.2
χ^2^		2.042	0.622	2.158	1.829	0.679	1.277	1.255	0.938	1.873
*p*		0.111	0.602	0.096	0.145	0.566	0.285	0.292	0.424	0.137
Second trimester										
S-LFI_1_	34 (23.9)	28.2 ± 3.1	157.0 ± 4.4	55.1 ± 5.5	22.3 ± 2.2	39.8 ± 3.5	23.9 ± 2.4	47.7 ± 4.7	113.2 ± 11.6	77.4 ± 6.9
S-LFI_2_	38 (26.8)	28.2 ± 3.6	157.4 ± 4.6	54.4 ± 4.6	22.0 ± 2.0	40.4 ± 3.7	22.6 ± 2.7	49.3 ± 5.1	114.2 ± 11.5	76.8 ± 6.5
S-HFI_1_	34 (23.9)	29.2 ± 3.2	156.9 ± 5.0	52.6 ± 5.2	21.4 ± 1.7	39.3 ± 3.1	24.5 ± 25	51.2 ± 6.7	112.1 ± 7.4	76.3 ± 6.2
S-HFI_2_	36 (25.4)	28.3 ± 3.4	156.3 ± 4.3	53.4 ± 4.9	21.8 ± 2.0	40.3 ± 3.7	23.7 ± 2.7	52.1 ± 7.0	112.3 ± 11.6	76.9 ± 7.3
S-Total	142	28.5 ± 3.3	156.9 ± 4.6	53.9 ± 5.1	21.9 ± 2.0	40.0 ± 3.5	23.6 ± 2.6	50.0 ± 6.1	113.0 ± 10.6	76.8 ± 6.7
χ^2^		0.760	0.361	1.626	1.400	0.680	3.439	4.209	0.287	0.141
*p*		0.518	0.781	0.186	0.246	0.566	0.019 *	0.007 *	0.835	0.935
Third trimester										
T-LFI_1_	35 (24.6)	28.8 ± 3.3	156.5 ± 5.1	60.6 ± 6.4	24.5 ± 2.1	40.6 ± 2.2	27.4 ± 2.4	49.6 ± 4.8	119.1 ± 9.1	72.9 ± 7.
T-LFI_2_	36 (25.4)	28.3 ± 3.2	156.4 ± 4.4	58.9 ± 6.5	24.1 ± 2.6	40.2 ± 2.5	27.9 ± 2.1	50.6 ± 5.5	115.2 ± 10.1	74.2 ± 7.7
T-HFI_1_	36 (25.4)	28.7 ± 3.6	156.7 ± 4.9	59.0 ± 6.9	24.0 ± 2.5	40.8 ± 2.2	27.0 ± 2.9	49.6 ± 6.1	116.6 ± 12.1	74.6 ± 6.9
T-HFI_2_	35 (24.6)	28.1 ± 3.2	158.0 ± 3.8	58.1 ± 5.1	23.3 ± 2.0	41.6 ± 2.2	28.4 ± 2.8	51.5 ± 3.3	112.7 ± 8.0	73.1 ± 5.9
T-Total	142	28.5 ± 3.3	157.0 ± 4.6	59.2 ± 6.3	24.0 ± 2.3	40.8 ± 2.3	27.7 ± 2.6	50.3 ± 5.0	115.9 ± 10.1	73.7 ± 7.0
χ^2^		0.321	0.907	0.969	1.803	2.662	1.875	1.175	2.534	0.507
*p*		0.811	0.440	0.409	0.150	0.051	0.137	0.321	0.059	0.678

Note: ^a^ Values presented as numbers (percentage) and compared using the chi-squared test. ^b^ Values presented as mean ± SD and compared using one-way ANOVA. *, means that there was significant difference as the *p*-value was less than 0.05. SD: standard deviation; BMI: body mass index. TFI: total fluid intake. AI represents recommendations for adequate intake level. The AI recommendation for TFI levels for pregnant women set by the Chinese nutrition society is 1.7 L. F-LFI_1_: first trimester-low fluid intake 1; F-LFI_2_: first trimester-low fluid intake 2; F-HFI_1_: first trimester-high fluid intake 1; F-HFI_2_: first trimester-high fluid intake 2. S-LFI_1_: second trimester-low fluid intake 1; S-LFI_2_: second trimester-low fluid intake 2; S-HFI_1_: second trimester-high fluid intake 1; S-HFI_2_: second trimester-high fluid intake 2. T-LFI_1_: third trimester-low fluid intake 1; T-LFI_2_: third trimester-low fluid intake 2; T-HFI_1_: third trimester-high fluid intake 1; and T-HFI_2_: third trimester-high fluid intake 2.

**Table 3:** Composition of fluid intake of participants in different trimesters with different TFI levels.

	*N* ^a^	Daily TFI (mL) ^b^	Percentage Meeting Chinese Water AI Level (%) ^b^	Sources					
				Plain Water		Dairy Products		SSBs	
				Amount (mL) ^b^	Percent (%) ^b^	Amount (mL) ^b^	Percent (%) ^b^	Amount (mL) ^b^	Percent (%) ^b^
First trimester									
F-LFI_1_	38	1210 (69)	0 (0.0)	1101 (104)	92.6 (7.2)	54 (64)	4.5 (5.5)	29 (57)	2.4 (4.8)
F-LFI_2_	36	1299 (37)	0 (0.0)	1211 (71)	92.8 (3.4)	60 (53)	4.6 (4.0)	29 (71)	2.2 (5.6)
F-HFI_1_	33	1375 (54)	0 (0.0)	1242 (95)	90.2 (6.3)	83 (56)	6.0 (4.1)	54 (92)	2.0 (6.7)
F-HFI_2_	35	1502 (54)	0 (0.0)	1242 (95)	90.9 (8.9)	61 (66)	4.0 (4.2)	68 (33)	2.2 (5.5)
F-Total	142	1336 (83)	0 (0.0)	1364 (126)	92.0 (6.6)	61 (60)	4.6 (4.6)	29 (73)	2.2 (5.9)
χ^2^		132.134	0.000	96.462	4.374	5.827	4.790	3.079	1.467
*p*		<0.001 *	1.000	<0.001 *	0.224	0.120	0.188	0.380	0.690
Second trimester									
S-LFI_1_	34	1399 (110)	0 (0.0)	1252 (84)	95.1 (4.6)	56 (59)	4.1 (4.2)	0 (2.8)	0.0 (2.1)
S-LFI_2_	38	1442 (37)	0 (0.0)	1351 (79)	93.9 (4.5)	52 (56)	3.6 (4.0)	26 (46)	1.8 (3.2)
S-HFI_1_	34	1514 (37)	0 (0.0)	1435 (104)	94.3 (5.6)	64 (55)	4.2 (3.6)	29 (64)	1.9 (4.3)
S-HFI_2_	36	1599 (80)	4 (11.1)	1510 (109)	93.6 (5.4)	65 (54)	3.9 (3.4)	32 (70)	1.9 (4.1)
S-Total	142	1477 (59)	4 (2.8)	1379 (158)	94.2 (4.8)	57 (56)	4.0 (3.7)	23 (64)	1.5 (4.0)
χ^2^		132.150	12.034	99.640	99.640	3.078	3.078	10.298	10.298
*p*		<0.001 *	0.007 *	<0.001 *	<0.001 *	0.380	0.380	0.016 *	0.016 *
Third trimester									
T-LFI_1_	35	1433 (134)	0 (0.0)	1320 (136)	93.7 (3.8)	56 (59)	4.4 (4.4)	30 (49)	2.1 (3.2)
T-LFI_2_	36	1552 (71)	0 (0.0)	1456 (69)	93.7 (4.1)	58 (53)	3.7 (3.4)	25 (75)	1.6 (4.8)
T-HFI_1_	36	1610 (27)	0 (0.0)	1506 (95)	93.0 (5.6)	48 (61)	3.0 (3.7)	29 (77)	1.8 (4.8)
T-HFI_2_	35	1729 (84)	21 (60.0)	1577 (104)	91.6 (4.8)	64 (61)	3.8 (3.3)	54 (70)	3.1 (4.0)
T-Total	142	1584 (139)	21 (14.8)	1437 (151)	93.4 (5.0)	59 (58)	3.7 (3.6)	31 (75)	2.1 (4.7)
χ^2^		132.198	75.342	102.567	3.488	1.487	1.734	7.417	4.789
*p*		<0.001 *	<0.001 *	<0.001 *	0.322	0.685	0.629	0.060	0.188
*F* ^#^		134.155	29.840	149.534	23.254	2.851	12.684	6.171	9.535
*p*		<0.001 *	<0.001 *	<0.001 *	<0.001 *	0.240	0.002 *	0.046 *	0.009 *

Note: ^a^ values presented as number and compared using the chi-squared test; ^b^ values presented as median (quartile ranges) and compared using the Kruskal–Wallis test. ^#^: *F*-value means the statistical test value calculated using Friedman’s test when the number of cases was 142 and the degrees of freedom was 2. * values mean there existed significant differences as a *p*-value was less than 0.05. TFI means total fluid intake; SSBs means sugar-sweetened beverages. AI represents recommendations for adequate intake level. The AI recommendation for TFI levels for pregnant women set by the Chinese nutrition society is 1.7 L.

**Table 4:** Urine indexes for participants with different TFI levels in different trimesters of pregnancy.

	*N* ^a^	Urine Osmolality (mmol/L) ^b^	Hydration Status (n, %) ^c^			USG ^b^	Urine pH ^b^	Urea (mmol/L) ^b^	Urine Creatinine (mmol/L) ^b^	Uric Acid (mmol/L) ^b^
			Optimal Hydrated Status	Normal Hydrated Status	Dehydrated Status					
First trimester										
F-LFI_1_	38	788 (124)	2 (1.4%)	15 (10.6%)	16 (11.3%)	1.020 (0.019)	6.0 (1.0)	4.1 (1.0)	55.9 (13.8)	261.0 (80.0)
F-LFI_2_	36	674 (147)	3 (2.1%)	30 (21.1%)	5 (3.5%)	1.021 (0.010)	6.3 (1.5)	4.1 (1.4)	59.8 (10.2)	292.5 (107.3)
F-HFI_1_	33	612 (314)	13 (9.2%)	19 (13.4%)	4 (2.8%)	1.015 (0.011)	6.0 (1.3)	3.9 (1.1)	57.4 (8.7)	266.0 (68.6)
F-HFI_2_	35	456 (175)	22 (15.5%)	12 (8.5%)	1 (0.7%)	1.010 (0.005)	6.5 (1.0)	4.0 (1.5)	55.7 (5.7)	258.0 (93.0)
F-Total	142	666 (296)	40 (28.2%)	76 (53.5%)	26 (18.3%)	1.015 (0.013)	6.0 (1.3)	4.0 (1.1)	57.4 (10.4)	265.0 (83.4)
χ^2^		46.197		56.987		9.591	12.138	2.018	11.295	1.382
*p*		<0.001 *		<0.001 *		0.022 *	0.007 *	0.569	0.010 *	0.710
Second trimester										
S-LFI_1_	34	764 (128)	3 (2.1%)	26 (18.3%)	5 (3.5%)	1.020 (0.016)	6.0 (1.4)	3.7 (1.4)	58.3 (8.8)	248.5 (51.3)
S-LFI_2_	38	712 (126)	4 (2.8%)	31 (21.8%)	3 (2.1%)	1.022 (0.010)	6.0 (1.3)	3.7 (1.3)	53.5 (10.6)	252.6 (88.5)
S-HFI_1_	34	665 (271)	10 (7.0%)	23 (9.2%)	1 (0.7%)	1.015 (0.011)	6.0 (1.2)	3.8 (1.3)	55.4 (11.4)	165.0 (100.4)
S-HFI_2_	36	512 (253)	17 (12.0%)	19 (13.4%)	0 (0.0%)	1.015 (0.010)	6.3 (1.1)	4.0 (1.3)	58.9 (9.7)	245.5 (90.4)
S-Total	142	672 (256)	34 (23.9%)	99 (69.7%)	9 (6.3%)	1.020 (0.013)	6.0 (1.0)	3.9 (1.4)	56.5 (10.5)	254.0 (83.9)
χ^2^		26.728		23.970		8.847	4.084	3.242	4.285	0.988
*p*		<0.001 *		<0.001 *		0.031 *	0.252	0.356	0.232	0.804
Third trimester										
T-LFI_1_	35	675 (166)	1 (0.7%)	33 (23.2%)	1 (0.7%)	1.020 (0.015)	6.0 (1.5)	4.1 (1.2)	59.4 (14.1)	274.0 (93.3)
T-LFI_2_	36	646 (180)	3 (2.1%)	32 (22.5%)	1 (0.7%)	1.017 (0.013)	6.3 (0.9)	3.8 (1.2)	57.1 (9.9)	281.5 (64.0)
T-HFI_1_	36	665 (149)	4 (2.8%)	32 (22.5%)	0 (0.0%)	1.020 (0.016)	6.0 (1.3)	4.0 (1.1)	56.6 (14.3)	260.0 (120.5)
T-HFI_2_	35	521 (349)	16 (11.3%)	19 (13.4%)	0 (0.0%)	1.010 (0.015)	6.5 (1.0)	4.0 (1.3)	53.3 (5.9)	235.0 (66.0)
T-Total	142	659 (185)	24 (16.9%)	116 (81.7%)	2 (1.4%)	1.015 (0.013)	6.01 (1.0)	4.0 (1.1)	56.0 (10.6)	260.5 (79.5)
χ^2^		15.298		29.909		2.578	6.623	1.744	12.995	8.503
*p*		0.002 *		<0.001 *		0.461	0.085	0.627	0.005 *	0.037 *
*F* ^#^		2.898		1.047		0.841	0.559	1.441	1.310	3.858
*p*		0.235		0.593		0.657	0.756	0.487	0.519	0.145

Note: ^a^ values presented as number; ^b^ values presented as median (quartile ranges) and compared using the Kruskal–Wallis test; ^c^ values presented as n (percentage) and compared using the chi-squared test. ^#^: *F*-value means the statistical test value calculated using Friedman’s test when the number of cases was 142 and the degrees of freedom was 2. * values mean there existed significant differences as a *p*-value of less than 0.05 was considered significant. USG means urine-specific gravity.

**Table 5:** Blood indexes of participants with different TFI levels in different trimesters of pregnancy.

	Blood Glucose (mmol/L) ^a^	Blood Lipid (mmol/L)				Leukocyte Count (10^9^/L) ^a^	Red Blood Cell Count (10^12^/L) ^a^	Hemoglobin Concentration (g/L) ^a^	Hematocrit (%) ^a^	Mean Red Blood Cell Volume (fL) ^a^	Mean Hemoglobin Content (pg) ^a^	Platelet Count (10^9^/L) ^a^	Total Serum Protein (g/L) ^a^	Total Serum Bilirubin (μmoI/L) ^a^	Lymphocyte Count ^a^ (10^9^/L)
		TG ^a^	TC ^a^	HDL ^a^	LDL ^a^										
First trimester															
F-LFI_1_	5.4 (0.5)	0.67 (0.80)	5.02 (1.02)	2.10 (0.98)	3.43 (3.36)	6.1 (2.1)	4.6 (0.5)	134.0 (13.0)	40.1 (3.8)	87.0 (8.3)	327.0 (17.5)	266.0 (86.0)	75.5 (2.8)	9.7 (6.5)	32.7 (10.3)
F-LFI_2_	5.3 (0.9)	0.91 (0.51)	4.75 (0.98)	1.67 (0.69)	2.97 (1.01)	6.4 (2.2)	4.6 (0.5)	132.5 (20.0)	40.1 (3.8)	89.3 (15.5)	325.0 (18.5)	280.0 (66.0)	76.5 (3.4)	9.1 (6.6)	32.2 (10.4)
F-HFI_1_	5.3 (0.9)	0.73 (0.49)	4.87 (1.34)	1.82 (0.97)	2.93 (1.54)	6.2 (1.8)	4.7 (0.7)	135.0 (16.0)	41.1 (3.0)	87.7 (9.9)	324.5 (12.0)	288.5 (100.0)	76.1 (4.5)	9.0 (5.6)	34.0 (12.6)
F-HFI_2_	5.2 (0.6)	0.90 (0.60)	4.82 (1.24)	1.70 (0.41)	2.72 (0.95)	6.3 (2.0)	4.6 (0.5)	132.0 (18.0)	40.3 (4.4)	87.2 (10.6)	326.0 (15.0)	281.0 (87.0)	75.0 (4.1)	8.8 (5.7)	31.6 (11.1)
F-Total	5.3 (0.7)	0.85 (0.59)	4.83 (1.15)	1.80 (0.82)	2.98 (1.37)	6.3 (1.9)	4.6 (0.5)	133.0 (14.0)	40.5 (3.5)	87.5 (10.0)	325.0 (16.0)	281.0 (81.0)	76.0 (4.0)	9.2 (5.8)	32.6 (11.1)
χ^2^	1.862	9.264	0.971	5.270	10.005	0.623	2.109	1.359	3.437	0.500	0.164	1.133	5.128	1.145	1.122
*p*	0.602	0.026 *	0.808	0.153	0.019 *	0.891	0.550	0.715	0.329	0.919	0.983	0.769	0.163	0.766	0.772
Second trimester															
S-LFI_1_	5.0 (0.5)	0.79 (0.68)	4.79 (0.89)	1.72 (0.45)	2.63 (0.67)	6.5 (2.2)	4.6 (0.5)	132.0 (12.0)	40.5 (3.1)	87.2 (4.9)	326.0 (18.0)	276.0 (81.0)	74.2 (4.6)	10.4 (5.2)	35.3 (11.6)
S-LFI_2_	5.2 (0.8)	0.89 (0.38)	4.86 (1.33)	1.78 (1.12)	2.87 (1.05)	6.0 (2.2)	4.7 (0.6)	135.0 (20.0)	41.4 (3.8)	89.5 (8.7)	327.0 (14.0)	300.5 (94.0)	75.6 (4.1)	10.5 (5.8)	33.4 (11.7)
S-HFI_1_	5.3 (0.8)	0.78 (0.43)	4/60 (1.78)	1.69 (0.66)	2.52 (1.24)	6.2 (2.0)	4.7 (0.6)	131.5 (13.0)	40.6 (3.8)	87.1 (7.1)	327.0 (18.0)	294.5 (62.0)	75.4 (4.0)	8.5 (3.5)	34.6 (8.2)
S-HFI_2_	5.3 (0.6)	0.80 (0.47)	5.01 (0.98)	1.72 (0.82)	2.80 (0.86)	6.1 (2.0)	4l6 (0.8)	129.0 (12.0)	39.7 (4.6)	87.2 (10.7)	322.5 (15.0)	276.0 (83.0)	75.5 (4.2)	8.8 (4.7)	34.0 (10.1)
S-Total	5.2 (0.6)	0.83 (0.47)	4.80 (1.07)	1.71 (0.60)	2.75 (0.87)	6.1 (2.0)	4.6 (0.5)	132.0 (14.0)	40.7 (4.0)	88.0 (7.5)	325.0 (18.0)	287.0 (78.0)	75.3 (4.0)	9.3 (4.8)	34.1 (9.5)
χ^2^	6.289	5.642	2.340	1.756	2.875	1.065	1.577	3.842	1.113	1.300	4.601	2.121	2.525	5.660	0.990
*p*	0.098	0.130	0.505	0.625	0.411	0.785	0.665	0.279	0.774	0.729	0.203	0.548	0.471	0.129	0.804
Third trimester															
T-LFI_1_	3.6 (1.0)	0.76 (0.55)	5.06 (1.13)	1.78 (0.68)	2.81 (1.22)	6.1 (1.8)	4.8 (0.9)	133.0 (15.0)	40.5 (3.9)	86.9 (10.6)	322.0 (17.0)	299.0 (103.0)	75.0 (4.1)	9.7 (6.0)	35.5 (10.1)
T-LFI_2_	3.7 (1.5)	0.90 (0.74)	4.76 (1.35)	1.75 (0.57)	2.73 (0.89)	6.7 (2.2)	4.7 (0.8)	135.5 (18.0)	40.6 (4.6)	87.6 (13.0)	323.0 (20.5)	301.5 (83.0)	76.6 (4.6)	9.4 (5.4)	35.7 (9.0)
T-HFI_1_	3.5 (0.7)	0.93 (0.59)	5.14 (1.41)	1.80 (0.65)	2.91 (1.00)	5.9 (1.9)	4.7 (0.4)	134.0 (14.0)	40.8 (3.1)	89.2 (5.6)	328.5 (15.8)	288.0 (110.0)	75.5 (4.5)	9.4 (7.3)	34.6 (10.2)
T-HFI_2_	3.8 (1.4)	0.86 (0.53)	4.74 (1.17)	1.64 (0.66)	2.66 (1.02)	6.2 (1.4)	4.6 (0.4)	134.0 (13.0)	40.3 (3.1)	89.8 (6.5)	324.0 (17.0)	294.0 (115.0)	76.3 (4.1)	8.2 (3.7)	33.9 (14.9)
T-Total	3.7 (1.3)	0.85 (0.51)	4.98 (1.20)	1.87 (0.67)	2.83 (0.96)	6.3 (1.7)	4.6 (0.6)	134.0 (14.0)	40.5 (3.4)	88.0 (7.8)	325.0 (17.0)	293.5 (102.0)	75.8 (4.2)	9.3 (4.8)	35.1 (10.9)
χ^2^	3.800	2.568	4.657	1.985	2.063	4.715	5.662	0.247	1.083	5.524	3.014	2.675	6.039	1.565	1.936
*p*	0.284	0.463	0.199	0.576	0.559	0.194	0.129	0.970	0.781	0.137	0.389	0.444	0.110	0.667	0.586
*F* ^#^	122.187	1.862	1.422	0.473	8.646	0.042	0.131	0.677	1.975	1.706	1.562	1.172	0.217	0.011	6.233
*p*	<0.001 *	0.394	0.491	0.789	0.013 *	0.979	0.937	0.713	0.372	0.426	0.458	0.557	0.897	0.995	0.044 *

Note: ^a^ Values presented as median (quartile ranges) and compared using the Kruskal–Wallis test. ^#^: *F*-value means the statistical test value calculated using Friedman’s test when the number of cases was 142 and the degrees of freedom was 2. * Values mean there existed significant differences as a *p*-value of less than 0.05 was considered significant. TC means total cholesterol. TG means triglyceride. LDL means low-density lipoprotein. HDL means high-density lipoprotein.

**Table 6:** Correlations between fluid intake, hydration status, urine biomarkers, and blood biomarkers during different trimesters.

	TFI		Plain Water		Dairy Products		SSBs	
	*r*	*p*	*r*	*p*	*r*	*p*	*r*	*p*
The first trimester								
Urine osmolality (mOsm/kg)	−0.596	<0.001 *	−0.312	<0.001 *	0.028	0.737	−0.012	0.884
Hydration status	−0.570	<0.001 *	−0.716	<0.001 *	−0.238	0.004 *	−0.052	0.536
USG	−0.180	0.033 *	−0.167	0.048 *	−0.012	0.886	0.128	0.131
Urine pH	0.132	0.120	0.216	0.010 *	−0.078	0.359	−0.058	0.495
Urine creatinine (mmol/L)	−0.063	0.460	−0.021	0.806	−0.064	0.446	0.045	0.597
TG (mmol/L)	0.112	0.185	0.200	0.017 *	−0.128	0.131	−0.036	0.668
LDL (mmol/L)	−0.261	0.002 *	−0.312	<0.001 *	0.028	0.737	−0.012	0.884
The second trimester								
Urine osmolality (mOsm/kg)	−0.439	<0.001 *	−0.418	<0.001 *	−0.043	0.610	−0.023	0.782
Hydration status	−0.410	<0.001 *	−0.349	<0.001 *	−0.094	0.266	−0.068	0.421
USG	−0.071	0.402	−0.083	0.329	−0.030	0.721	−0.057	0.504
Urine pH	−0.054	0.530	−0.017	0.841	0.060	0.483	0.078	0.357
The third trimester								
Urine osmolality (mOsm/kg)	−0.295	<0.001 *	−0.213	0.011 *	0.030	0.721	−0.124	0.140
Hydration status	−0.444	<0.001 *	−0.368	<0.001 *	−0.021	0.800	−0.137	0.103
Urine creatinine (mmol/L)	−0.252	0.002 *	−0.253	0.002 *	0.145	0.086	−0.109	0.196
Uric acid (mmol/L)	−0.230	0.006 *	−0.269	0.002 *	−0.026	0.760	0.014	0.865

Note: Spearman’s correlation coefficients were used to analyze the intensity of the correlations among fluid intake behaviors and night urination. *, means that there was statistically significant correlations, as the *p*-value was less than 0.05. TFI means total fluid intake. SSBs means sugar-sweetened beverages. USG means urine-specific gravity. TG means triglyceride. LDL means low-density lipoprotein.

## Data Availability

The data are available from the corresponding authors upon reasonable request.

## Abbreviations

BMI	Body mass index
SSB	Sugar-sweetened beverage
TFI	Total fluid intake
USG	Urine-specific gravity
